# Anti-gout Medications and Risk of Cardiovascular Disease: A Nested Case-Control Study

**DOI:** 10.3389/fmed.2021.739680

**Published:** 2021-10-18

**Authors:** Tsung-Ju Chuang, Yu-Hsun Wang, James Cheng-Chung Wei, Chih-Jung Yeh

**Affiliations:** ^1^Division of Endocrinology and Metabolism, Department of Internal Medicine, Taichung Armed Forces General Hospital, Taichung, National Defense Medical Center, Taipei, Taiwan; ^2^School of Public Health, Chung Shan Medical University, Taichung, Taiwan; ^3^Department of Medical Research, Chung Shan Medical University Hospital, Taichung, Taiwan; ^4^Department of Allergy, Immunology and Rheumatology, Chung Shan Medical University Hospital, Taichung, Taiwan; ^5^Institute of Medicine, Chung Shan Medical University, Taichung, Taiwan; ^6^Graduate Institute of Integrated Medicine, China Medical University, Taichung, Taiwan

**Keywords:** gout, cardiovascular disease, drug adherence, uric acid, urate-lowering therapy (ULT)

## Abstract

**Introduction:** Gout is the leading cause of inflammatory arthritis and is also correlated with multiple comorbidities, including cardiovascular disease (CVD), whose future risk can be lowered by urate-lowering therapy (ULT) in gout patients. It is, however, still not clear whether its effect is associated with the days of usage and the adherence rate of ULT.

**Methods:** Data were collected from Taiwan's National Health Insurance Research Database. The study period was from 1999/1/1 to 2013/12/31. In addition, patients with newly diagnosed gout from 2000 to 2012 and usage of antigout preparations (allopurinol or benzbromarone) within half a year among age ≥20 years old were enrolled in the study. The outcome of interest is CVD. New diagnosis of CVD after half a year of diagnosis of gout was included in the CVD group. Moreover, conditional logistic regression was used to evaluate the odds ratio of CVD in relation to the days of usage and to the adherence rate of ULT after the adjustment for potentially confounding variables.

**Results:** A total of 3,706 gout patients with and without CVD have been included in the final analysis after a 1:1 propensity score that matched for age, sex, comorbidities, aspirin, and statin. The days of usage of allopurinol was <180 days and benzbromarone, in its turn, presupposed a higher risk of CVD. The adherence rate of allopurinol and benzbromarone at ≥ 0.7 both have a lower CVD risk: allopurinol (adjusted OR: 0.66 95% CI: 0.46–0.96), benzbromarone (adjusted OR: 0.68 95% CI: 0.50–0.91). The subgroup analysis revealed an adherence rate of ≥0.7 of ULT with a lower CVD was only found to be present in males and at age <65. Furthermore, the correlations were more pronounced in the ischemic heart disease subgroup than in the cerebrovascular disease group.

**Conclusion:** This study reveals that gout patients taking ULT (allopurinol and benzbromarone) with an adherence rate of ≥0.7 are at a lower risk of developing CVD, especially with a younger age (<65) and if they are male. On top of this, the benefit is more pronounced in ischemic heart disease. Despite further prospective trials needing to be warranted to confirm our findings, health care providers may, bearing these conclusions in mind, emphasize the importance of adherence to ULT in gout patients.

## Introduction

Gout is the main cause of inflammatory arthritis, usually affecting men, and is characterized by intermittent and sudden onset of intense inflammation (1), and it is characterized by hyperuricemia, serum, or plasma uric acid concentration of >6.8 mg/dL, close to the dissolution limit of uric acid (2). Gout may present itself through different manifestations of diseases, such as recurrent episodes of inflammatory arthritis, chronic joint disease, accumulation of urate crystals in the form of stone deposits, and uric acid (UA) kidney stones (3). In addition, gout is also associated with various comorbidities such as diabetes, hypertension, chronic kidney disease, and cardiovascular disease (CVD) (4).

The treatment of gout includes the treatment of acute attacks and chronic phases.

In the acute phase, several types of anti-inflammatory drugs are effective, including colchicine, non-steroidal anti-inflammatory drugs, systemic and intra-articular glucocorticoids, and biological agents that inhibit the action of interleukin-1β (3, 5).

In the chronic stage, long-term use of urate-lowering therapy (ULT) can prevent gout flare, tophi formation, and related comorbidities (6). ULT usually includes xanthine oxidase inhibitors and uricosuric drugs, used to make serum uric acid levels <6 mg/dL, which is significantly lower than the uric acid solubility limit (6–8). The ULT should be selected according to the patient's hepatic and renal functions and their comorbidities.

Common xanthine oxidase inhibitors include allopurinol and febuxostat, which reduce the synthesis of UA by inhibiting the activity of xanthine oxidase (2). In adults, the starting dose of allopurinol is 50–100 mg/d, which can be increased up to a maximum dose of 600 mg/d until the UA target is reached. In addition, the recommended dose for chronic kidney disease (CKD) stages 3–4 is 50–100 mg/d, and CKD stage five patients are contraindicated (9). On the other hand, febuxostat is a selective xanthine oxidase inhibitor. The initial dose is 20–40 mg/d, and it can be titrated up to a maximum of 80 mg/d until it reaches the serum UA target, which proves that it is effective in patients with impaired renal function, because it is mainly cleared by the liver (10).

The uricosuric drugs include probenecid, sulfinpyrazone, and benzbromarone.

Probenecid can be started at 250 mg twice daily and may be increased to a maximum of 2 g/day. In addition, probenecid should be avoided in patients with eGFR <30 mL/min (11, 12). In its turn, sulfinpyrazone starts at a dose of 50 mg per day and can be increased to the maximum effective dose (800 mg/day) and should be avoided in patients with CKD or history of UA kidney stones (13). Finally, the starting dose of benzbromarone is 25–50 mg/d in adults and should be adjusted to 75 mg/d or 100 mg/d according to the serum UA level and is contraindicated in patients with severe renal dysfunction or UA nephrolithiasis (14). Moreover, its greatest adverse reaction is hepatotoxicity (15).

CVD is the leading cause of death worldwide, which may be due to the increased incidence of chronic diseases such as hypertension, hyperlipidemia, obesity, and diabetes (16, 17). Apart from the traditional risk factors, gout and hyperuricemia were also found to be associated with CVD and its mortality (17–19). Moreover, several studies investigated the associations between ULT and CVD risk and mortality. Chen et al. found that hyperuricemia patients who received ULT had significantly lower all-cause mortality compared to the matched hyperuricemia patients who did not receive ULT. However, the CVD mortality risk between ULT users and non-users was not statistically significant due to a small number of mortality events (20). In addition, recent large-sample analyses further confirm that gout patients receiving ULT had a lower risk of further CAD and stroke (21, 22). It is, however, still not known whether the usage days and adherence rates of ULT are correlated with further risk of CAD.

In the study, we have therefore enrolled gout patients from the Taiwan Nation Health Insurance Research Database (NHIRD) in order to investigate the correlations between ULT (allopurinol and benzbromarone) and risk of CVD in gout patients, specifically focusing on the days of usage and on the adherence rates according to age and gender.

## Methods

### Data Source

The data were collected from the Longitudinal Health Insurance Database (LHID) 2000, which contains the clinical information of 1 million beneficiaries that were randomly selected from the NHIRD during the period ranging from 2000 to 2013 (23). The data of NHIRD was based on a single-payer government-run National Health Insurance program developed in Taiwan in 1995, which currently includes comprehensive health care data (hospitalization, emergency care, and medical visits) for almost all 23 million Taiwanese citizens, and the databases were used for research purposes (24). The International Statistical Classification of Diseases and Related Health Problems, 9th Revision, Clinical Modification (ICD-9-CM) is used for the physicians' diagnosis, and the data include patients' medical drug items, dosage, frequency of use, and the number of days. In addition, this information can be used to detect drug interactions and potential repeated medications when patients visit multiple hospitals. In its turn, the National Health Insurance (NHI) code corresponds to a code in the five-level Anatomical Therapeutic Chemical classification system, having been recommended by the World Health Organization for studies on drug utilization (25).

### Data Collection

The study period was from 1999/1/1 to 2013/12/31. We identified 1 million people from the database, of which we selected, based on the ICD-9-CM code, 274 patients with newly diagnosed gout from 2000 to 2012 undergoing ULT that included allopurinol (NHI code: M04AA01) or benzbromarone (NHI code: M04AB03), who used it within half a year, and who were ≥ 20 years of age, having excluded the subjects treated by probenecid and sulfinpyrazone due to the small number of cases. In this regard, subjects that were diagnosed with CVD (ICD-9-CM = 410–414, 430–438) before the date of first gout diagnosis were also excluded.

Individuals were divided into two groups, the CVD group and the non-CVD group, the former including individuals newly diagnosed with CVD followed by either ≥3 outpatient assessments or a single inpatient admission after more than half a year of diagnosis of gout. These two groups were, then, initially matched 1:1 according to age (±2 y/o), sex, and the diagnosis year of gout. After the initial match, these two groups also received a Propensity Score Matching (PSM) 1:1 according to age, sex, comorbidities, aspirin, and statin.

### Patient Subgroups

While the age of the patients was defined on the index date, 0 represented female and 1 represented male in relation to the sex variable. Furthermore, baseline comorbidities, which were diagnosed from three outpatient or one inpatient assessment 1 year before CVD diagnosis, included hypertension (ICD-9-CM = 401–405), hyperlipidemia (ICD-9-CM = 272.0–272.4), diabetes (DM) (ICD-9-CM = 250), chronic liver disease (CLD; ICD-9-CM = 571), chronic obstructive pulmonary disease (COPD; ICD-9-CM = 491, 492, and 496), and autoimmune disease (AD; ICD-9-CM = 710, 714, and 720). Finally, the use of aspirin or statin more than 30 days from the gout date to index date (CVD) was included in the baseline characteristics.

### Statistical Analysis

All analyses were performed using SAS version 9.1.3 for Windows (SAS Institute, Inc., Cary, NC) and *P*-values < 0.05 were considered statistically significant. Conditional logistic regression was used to evaluate the odds ratio (OR) of CVD in relation to the different ULT after due adjustment for potential confounding variables (age, sex, hypertension, hyperlipidemia, CLD, DM, COPD, AD, aspirin, statin, allopurinol, and benzbromarone). In addition, we analyzed the risks of CAD and stroke by three cumulative durations of ULT (<90 days, 90–180 days, or ≥ 180 days) and the adherence rate of therapy (<0.3, 0.3–0.6, or ≥ 0.7) relative to no-use of ULT in order to assess the dose–response relationship.

Kaplan–Meier analysis was applied to evaluate the cumulative incidence of CVD in the subgroups and log-rank test was used to test for significance. The subgroup analyses were, moreover, performed according to age (<65 years and ≥65 years), gender, and different CVD (ischemic heart disease or cerebrovascular disease). The absolute standardized differences (ASD) were analyzed in the before PSM and after PSM matched samples for baseline covariates comparing CVD to non-CVD subjects, and a standardized difference of 10% (or 0.1) ASD is equivalent to having a phi coefficient of 0.05 (indicating negligible correlation) for the correlation between the treatment group and the binary variable (26). Drug adherence rate was measured as the days of usage of ULT, which were divided according to the observational period.

## Results

**Figure 1:**
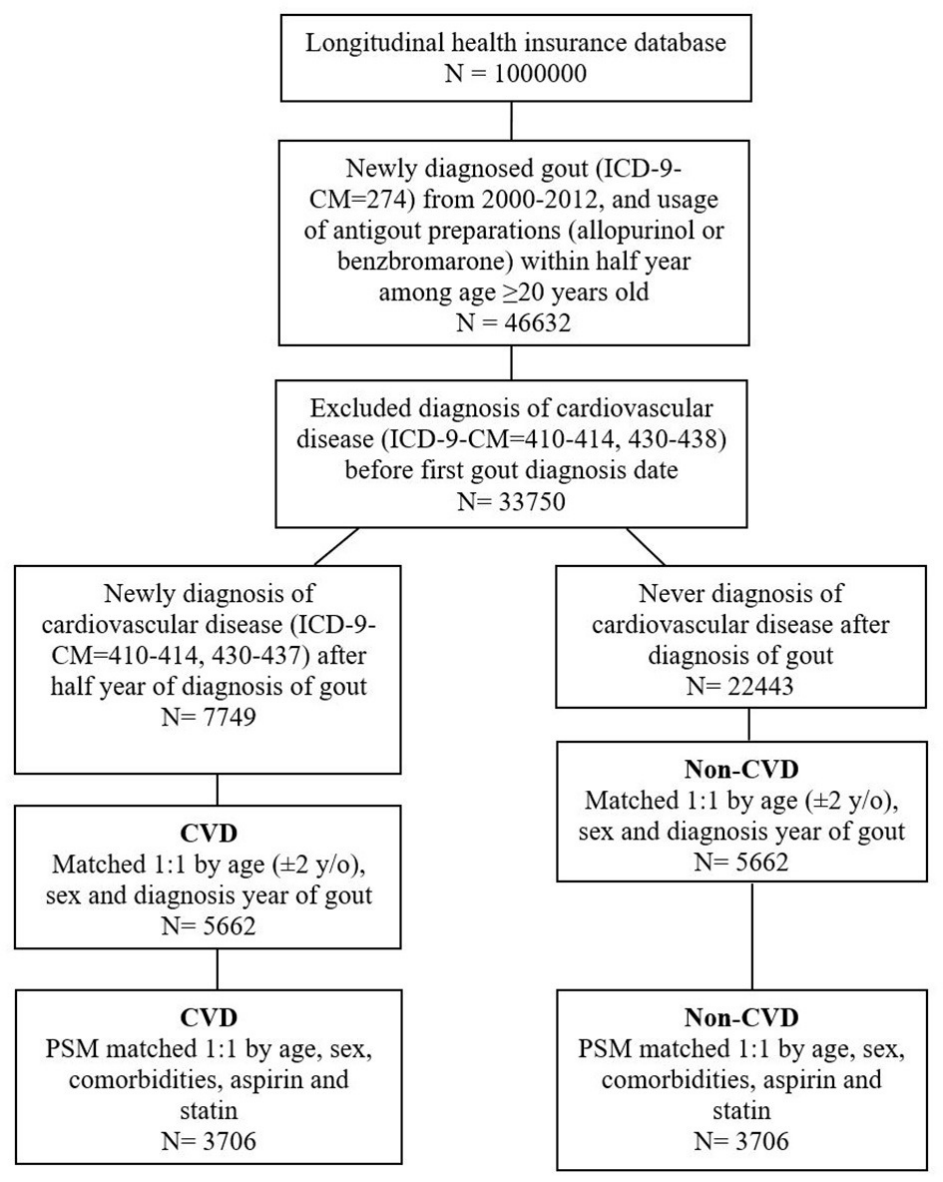
The study population selection protocol.

As shown in the flowchart in Figure 1, patients with and without CVD who were selected from the LHID 2000 and included in the final analysis were matched 1:1 by age, gender, and by the year of diagnosis of gout, each group consisting of 5,662 patients, of which remained 3,706 patients in each after PSM 1:1 by age, sex, comorbidities, aspirin, and statin.

In this regard, Table 1 shows the demographic characteristics of the CVD and non-CVD groups before PSM and after PSM. Before PSM, only age, sex, autoimmune disease, allopurinol, and benzbromarone had an ASD lower than 0.1, whereas all the comorbidities, aspirin, and statin after PSM showed to have an ASD lower than 0.1, which indicated a negligible correlation.

**Table 1:** Demographic characteristics.

	**Before PSM matched**					**After PSM matched**				
	**CVD (***N*** = 5,662)**		**Non-CVD (***N*** = 5,662)**			**CVD (***N*** = 3,706)**		**Non-CVD (***N*** = 3,706)**		
	***n***	**%**	***N***	**%**	**ASD**	***n***	**%**	***N***	**%**	**ASD**
Age					0.067					0.050
20–40	340	6.0	351	6.2		217	5.9	271	7.3	
40–65	3,607	63.7	3,799	67.1		2,333	63.0	2,247	60.6	
≥65	1,715	30.3	1,512	26.7		1,156	31.2	1,188	32.1	
Mean ± SD	58.4 ± 12		57.2 ± 11.7		0.101	58.5 ± 12.2		58.2 ± 12.3		0.021
Sex					0.000					
Female	1,527	27.0	1,527	27.0		1,001	27.0	1,014	27.4	
Male	4,135	73.0	4,135	73.0		2,705	73.0	2,692	72.6	
Hypertension	3,175	56.1	1,507	26.6	0.627	1,483	40.0	1,507	40.7	0.013
Hyperlipidemia	1,377	24.3	705	12.5	0.310	698	18.8	681	18.4	0.012
Chronic liver disease	470	8.3	303	5.4	0.117	256	6.9	275	7.4	0.020
Diabetes	1,120	19.8	575	10.2	0.272	563	15.2	552	14.9	0.008
COPD	245	4.3	113	2.0	0.134	95	2.6	112	3.0	0.028
Autoimmune disease	95	1.7	68	1.2	0.040	45	1.2	51	1.4	0.014
Statin	1,393	24.6	889	15.7	0.223	786	21.2	776	20.9	0.007
Aspirin	1,447	25.6	399	7.0	0.518	435	11.7	399	10.8	0.031
Allopurinol	2,620	46.3	2,453	43.3	0.059	1,681	45.4	1,630	44.0	0.028
Benzbromarone	4,581	80.9	4,453	78.6	0.056	3,008	81.2	2,954	79.7	0.037

*ASD, Absolute standardized difference; COPD, Chronic obstructive pulmonary disease*.

Table 2 shows the risk of CVD divided according to days of usage of allopurinol and benzbromarone. In model 1, after adjusting for confounding factors (age, sex, hypertension, hyperlipidemia, CLD, DM, COPD, AD, and allopurinol or benzbromarone), fewer days of usage of allopurinol has a higher risk of CVD (<90 days: OR, 1.18; 90–180 days: OR, 1.42) compared to the reference. In the benzbromarone group, fewer days of usage (<90 days) likewise showed a higher risk of CVD (OR, 1.32). The trend of risks is shown in model 2, being the same after adjusting for statin and aspirin.

**Table 2:** Logistic regression of risk of CVD divided by usage days of allopurinol and benzbromarone.

	***N***	**No. of CVD**	**cOR (95% C.I.)**	***p*** **-value**	**Model 1**		**Model 2**	
					**aOR (95% C.I.)**	***p*** **-value**	**aOR (95% C.I.)**	***p*** **-value**
Allopurinol								
None	4,101	2,025	Reference		Reference		Reference	
<90 days	2,193	1,113	1.06 (0.95–1.17)	0.299	1.18 (1.05–1.34)	0.008	1.18 (1.04–1.34)	0.008
90–180 days	427	237	1.28 (1.05–1.56)	0.016	1.42 (1.15–1.75)	0.001	1.42 (1.15–1.74)	0.001
≥180 days	691	331	0.94 (0.80–1.11)	0.473	1.05 (0.88–1.24)	0.606	1.04 (0.87–1.23)	0.675
Benzbromarone								
None	1,450	698	Reference		Reference		Reference	
<90 days	3,385	1,764	1.17 (1.04–1.33)	0.011	1.32 (1.14–1.53)	<0.001	1.32 (1.14–1.53)	<0.001
90–180 days	1,013	505	1.07 (0.91–1.26)	0.402	1.18 (0.99–1.41)	0.062	1.18 (0.98–,1.41)	0.074
≥180 days	1,564	739	0.97 (0.84–1.11)	0.626	1.05 (0.89–1.23)	0.546	1.04 (0.89–1.22)	0.622

*cOR, crude odds ratio; aOR, adjusted odds ratio*.

*Model 1: Adjusted for allopurinol, benzbromarone, age, sex, hypertension, hyperlipidemia, chronic liver disease, diabetes, COPD, and autoimmune disease*.

*Model 2: Adjusted for allopurinol, benzbromarone, age, sex, hypertension, hyperlipidemia, chronic liver disease, diabetes, COPD, autoimmune disease, statin, and aspirin*.

In Table 3, the conditional logistic regression of risk of CVD was divided into the adherence rate of both allopurinol and benzbromarone (<0.3, 0.3–0.6, ≥0.7), which, after adjusting for the confounding factors, statin and aspirin showed only an adherence rate of allopurinol and benzbromarone of ≥ 0.7 and had a lower risk of CVD (allopurinol: adjusted OR, 0.66, *P*: 0.03; benzbromarone: adjusted OR, 0.68, *P*: 0.01).

**Table 3:** Logistic regression of risk of CVD divided by adherence rate of allopurinol and benzbromarone.

	***N***	**No. of CVD**	**Crude OR (95% C.I.)**	***p*** **-value**	**Adjusted OR^†^(95% C.I.)**	***p*** **-value**
Adherence rate of allopurinol						
None	4,101	2,025	Reference		Reference	
<0.3	2,908	1,505	1.10 (1.00–1.21)	0.05004	1.15 (1.03–1.28)	0.017
0.3–0.6	266	125	0.91 (0.71–1.17)	0.451	0.95 (0.73–1.23)	0.702
≥0.7	137	51	0.61 (0.43–0.86)	0.006	0.66 (0.46–0.96)	0.030
Adherence rate of benzbromarone						
None	1,450	698	Reference		Reference	
<0.3	5,072	2,611	1.14 (1.02–1.28)	0.025	1.19 (1.03–1.37)	0.015
0.3–0.6	647	306	0.97 (0.80–1.16)	0.721	1.00 (0.82–1.23)	0.975
≥0.7	243	91	0.65 (0.49–0.85)	0.002	0.68 (0.50–0.91)	0.010

† *Adjusted for allopurinol, benzbromarone, age, sex, hypertension, hyperlipidemia, chronic liver disease, diabetes, COPD, autoimmune disease, statin, and aspirin*.

In Table 4, the adherence rate of allopurinol and benzbromarone was further divided in relation to age (<65, ≥65 years) and gender. Evidently, the logistic regression of risk of CVD in the younger group (<65 years) showed lower risks with the increased adherence of ULT, of which a rate of ≥ 0.7 had lower CVD risk in both allopurinol (adjusted OR, 0.62; 95% CI, 0.38–1.0) and benzbromarone (adjusted OR, 0.61; 95% CI, 0.41–0.90). All adherence rates of both allopurinol and benzbromarone, however, did not have a significantly lower risk of CVD in patients ≥ 65 years. In relation to gender, the larger adherence rate of both allopurinol and benzbromarone showed a lower risk of CVD in males as opposed to females. Specifically, the adjusted OR is 0.63 in allopurinol and 0.64 in benzbromarone with an adherence rate of ≥0.7.

**Table 4:** Logistic regression of risk of CVD divided by adherence rate of allopurinol and benzbromarone in different age and gender groups.

	***N***	**No. of CVD**	**Crude OR (95% C.I.)**	***p*** **-value**	**Adjusted OR^†^(95% C.I.)**	***p*** **-value**
**Age <65**						
Adherence rate of allopurinol						
None	2,749	1,382	Reference		Reference	
<0.3	2,064	1,064	1.05 (0.94–1.18)	0.38028	1.11 (0.97–1.26)	0.140
0.3–0.6	174	74	0.73 (0.54–1.00)	0.048	0.77 (0.55–1.06)	0.104
≥0.7	81	30	0.58 (0.37–0.92)	0.020	0.62 (0.38–1.00)	0.052
Adherence rate of benzbromarone						
None	982	466	Reference		Reference	
<0.3	3,547	1,837	1.19 (1.03–1.37)	0.016	1.20 (1.01–1.42)	0.037
0.3–0.6	401	197	1.07 (0.85–1.35)	0.572	1.05 (0.81–1.35)	0.707
≥0.7	138	50	0.63 (0.44–0.91)	0.014	0.61 (0.41–0.90)	0.013
**Age ≥65**						
Adherence rate of allopurinol						
None	1,352	643	Reference		Reference	
<0.3	844	441	1.21 (1.02–1.43)	0.032	1.24 (1.01–1.53)	0.039
0.3–0.6	92	51	1.37 (0.90–2.10)	0.145	1.45 (0.93–2.25)	0.099
≥0.7	56	21	0.66 (0.38–1.15)	0.142	0.72 (0.40–1.29)	0.270
Adherence rate of benzbromarone						
None	468	232	Reference		Reference	
<0.3	1,525	774	1.05 (0.85–1.29)	0.655	1.17 (0.91–1.51)	0.218
0.3–0.6	246	109	0.81 (0.59–1.10)	0.181	0.94 (0.66–1.34)	0.729
≥0.7	105	41	0.65 (0.42–1.00)	0.052	0.78 (0.49–1.24)	0.294
**Female**						
Adherence rate of allopurinol						
None	1,268	623	Reference		Reference	
<0.3	653	336	1.10 (0.91–1.33)	0.335	1.16 (0.91–1.48)	0.220
0.3–0.6	56	27	0.96 (0.56–1.65)	0.893	1.04 (0.59–1.82)	0.897
≥0.7	38	15	0.68 (0.35–1.31)	0.243	0.79 (0.39–1.59)	0.506
Adherence rate of benzbromarone						
None	415	203	Reference		Reference	
<0.3	1,339	680	1.08 (0.86–1.34)	0.506	1.18 (0.88–1.58)	0.262
0.3–0.6	192	91	0.94 (0.67–1.33)	0.728	1.05 (0.71–1.57)	0.793
≥0.7	69	27	0.67 (0.40–1.13)	0.133	0.77 (0.44–1.35)	0.362
**Male**						
Adherence rate of allopurinol						
None	2,833	1,402	Reference		Reference	
<0.3	2,255	1,169	1.10 (0.98–1.23)	0.096	1.14 (1.01–1.30)	0.040
0.3–0.6	210	98	0.89 (0.67–1.18)	0.430	0.92 (0.69–1.24)	0.589
≥0.7	99	36	0.58 (0.38–0.88)	0.011	0.63 (0.41–0.97)	0.037
Adherence rate of benzbromarone						
None	1,035	495	Reference		Reference	
<0.3	3,733	1,931	1.17 (1.02–1.34)	0.026	1.20 (1.02–1.41)	0.024
0.3–0.6	455	215	0.98 (0.78–1.22)	0.838	0.98 (0.77–1.25)	0.893
≥0.7	174	64	0.63 (0.46–0.88)	0.007	0.64 (0.45–0.91)	0.013

† *Adjusted for allopurinol, benzbromarone, age, sex, hypertension, hyperlipidemia, chronic liver disease, diabetes, COPD, autoimmune disease, statin, and aspirin*.

In Table 5, the CVD were further divided into ischemic heart disease and cerebrovascular disease. Regarding the former, allopurinol and benzbromarone both had a higher risk of ischemic heart disease with an adherence rate of <0.3 and a lower risk with an adherence rate of >0.7. In the group of cerebrovascular disease, however, only an adherence rate of benzbromarone of >0.7 proved to decrease the risk of cerebrovascular disease (*p*-value: 0.028).

**Table 5:** Sub-outcome of logistic regression of risk of CVD.

	**N**	**No. of CVD**	**Crude OR (95% C.I.)**	***p*** **-value**	**Adjusted OR^†^(95% C.I.)**	***p*** **-value**
**Ischemic heart disease**						
Adherence rate of allopurinol						
None	2,527	451	Reference		Reference	
<0.3	1,761	358	1.17 (1.01–1.37)	0.041	1.20 (1.00–1.43)	0.0497
0.3–0.6	170	29	0.95 (0.63–1.43)	0.795	1.02 (0.66–1.56)	0.930
≥0.7	93	7	0.37 (0.17–0.81)	0.013	0.46 (0.21–1.02)	0.057
Adherence rate of benzbromarone						
None	904	152	Reference		Reference	
<0.3	3,076	615	1.24 (1.02–1.50)	0.033	1.29 (1.02–1.62)	0.030
0.3–0.6	405	64	0.93 (0.68–1.28)	0.649	1.02 (0.72–1.45)	0.891
≥0.7	166	14	0.46 (0.26–0.81)	0.007	0.51 (0.28–0.93)	0.028
**Cerebrovascular disease**						
Adherence rate of allopurinol						
None	3,650	1,574	Reference		Reference	
<0.3	2,550	1,147	1.08 (0.97–1.19)	0.147	1.12 (0.99–1.26)	0.061
0.3–0.6	237	96	0.90 (0.69–1.17)	0.431	0.93 (0.70–1.22)	0.593
≥0.7	130	44	0.67 (0.47–0.98)	0.037	0.71 (0.48–1.05)	0.083
Adherence rate of benzbromarone						
None	1,298	546	Reference		Reference	
<0.3	4,457	1,996	1.12 (0.99–1.27)	0.083	1.16 (1.00–1.34)	0.058
0.3-0.6	583	242	0.98 (0.80–1.19)	0.821	1.00 (0.80–1.24)	0.977
≥0.7	229	77	0.70 (0.52–0.94)	0.017	0.71 (0.52–0.97)	0.034

† *Adjusted for allopurinol, benzbromarone, age, sex, hypertension, hyperlipidemia, chronic liver disease, diabetes, COPD, autoimmune disease, statin, and aspirin*.

## Discussions

Our study showed that only a high adherence rate of receiving ULT (allopurinol and benzbromarone) in gout patients was associated with lower risks of future CVD. In their turn, the subgroup analyses were able to reveal the trend is particularly significant in male patients and those of a younger age (<65 years).

The common use of ULT for gout patients is different according to each country.

A nationwide population study conducted from 2005 to 2010 in Taiwan reported that the prevalence of gout may be as high as 6.24%. However, only 22.93% of patients received ULT (27), among which 60.08% received uricosuric agents only (mostly benzbromarone), 28.54% received allopurinol, and 11.38% received both. In the present study, we have enrolled gout patients from 2000 to 2012, and like the previous epidemiology study, most patients use benzbromarone and Allopurinol for ULT. Additionally, the other recently commonly used xanthine oxidase inhibitor is febuxostat, which was approved by the U.S. Food and Drug Administration in 2009 and was not listed in Taiwan until 2012. Fewer patients in the study period used this drug and cannot, therefore, be enrolled in the analysis.

Previous studies have reported that elevated serum UA is closely related to coronary and carotid atherosclerosis (21, 28). Specifically, Mutluay et al. have reported that a higher serum UA in hypertensive subjects with normal renal function is an independent predictor of early atherosclerosis (29). The mechanisms linking elevated serum UA levels and gout to CVD comorbidities appear to be multifactorial, involving low-grade systemic inflammation and xanthine oxidase activity. Jacob George et al. have reported that UA acts as a pro-oxidant and induces endothelial dysfunction (30). In contrast, UA may also stimulate the renin-angiotensin system to further promote the growth of smooth blood vessel cells, as well as impaired arterial function and stiffening (31). Moreover, the correlation between hyperuricemia and systemic inflammatory markers (such as C-reactive protein) and tumor necrosis was also explored, showing a further contribution to CV damage (31); use of ULT may lower, as expected, serum UA, decreasing the risk of CVD. Hence, a past study has found that gout patients receiving ULT had a 32% lower risk of CVD compared with those not receiving ULT (22).

Dubreuil et al. have reported allopurinol can reduce all-cause and CVD mortality in the general population (32). Being treated with allopurinol over a 10-year period can reduce the risk of stroke (−50%) and cardiac event (−39%) in hypertensive patients, this being particularly significant in patients treated with a higher allopurinol dose (33). The dose-dependent trend was also found by Yen et al. their subgroup analyses indicating uricosuric agents among the ULT had a significant effect on the prevention of CVD development (21).

In the present study, we were further able to find that, instead, fewer days of usage of ULT (allopurinol < 180 days and benzbromarone < 90 days) increase the risks of CVD compared to the reference in gout patients, which can further emphasize the importance of the dose-dependent effect of ULT on CVD. The compliance and the adherence rate of ULT is important in gout patients and can influence the risk of CVD.

Likewise, a systematic review reported that less than half of gout patients in the real world adhere to treatment, and healthcare professionals in medication may emphasize the importance of monitoring, discussing, and supporting adherence to ULT (34, 35). The association between the adherence rate and the risk of CVD has not, however, been analyzed in the past, and we were able to find the adherence rate of ULT may significantly influence the risk of CVD in gout patients, in which only an adherence rate of ≥0.7 is able to reduce the risk of CVD both in the allopurinol (adjusted OR, 0.66) and benzbromarone (adjusted OR, 0.68) groups (Table 3).

The subgroup analysis revealed the protective effect of ULT on CVD was only seen in male patients and those of a younger age (<65). We can also find the trend at an older age and in females, but it is not significant. In this regard, the epidemiology study showed that the prevalence of gout is significantly higher in men. In men, the overall prevalence of gout can be 2.9-fold higher than in women, with this difference being observed in all ages and peaking at 7.3-fold from 30 to 34 years of age, showing to decrease thereafter (27). Gout in males is, therefore, an apparently younger onset disease; Li found that patients with a first diagnosis of gout at a younger age may be at an increased risk for CVD as opposed to those diagnosed later in life (36). This higher CVD risk in younger gout patients may explain the more significant effect in lowering CVD the higher adherence rate of ULT has in male patients and those of <65 years of age.

Moreover, the subgroup analysis also reported the high adherence to benzbromarone can reduce both incident ischemic heart disease and cerebrovascular disease (including ischemic and hemorrhagic stroke). However, the high adherence to allopurinol only have trend, proving not to play a significant role in reducing the risk of cerebrovascular disease. Apart from the fewer cases of allopurinol needing more subjects to achieve significance, the outcome was not totally consistent with the previous studies. Fu-Shun Yen et al. have reported ULT users among the patients with gout showed a lower risk of hospitalized ischemic stroke and all-cause mortality as opposed to non-users; the risks of hospitalized CAD, CV death, and hemorrhagic stroke do not, however, have obviously decrease (37). In its turn, Fu-Shun Yen et. Al. have also found ULT to be correlated with lower risks of incident CAD and stroke, with the subgroup analysis also revealing the effect is only significant in ischemic stroke as opposed to hemorrhagic stroke (21). Further prospective trials, specifically regarding the ischemic stroke, are further warranted to confirm our findings.

## Conclusions

The present study investigated the correlations between ULT (allopurinol and benzbromarone) and the risk of CVD in gout patients, showing that only a high adherence rate (≥0.7) of ULT can significantly reduce the risk of CVD, especially at a younger age (<65 years) and in male patients. Therefore, health care providers who prescribe gout medications may emphasize the importance of monitoring and supporting adherence to this therapy.

## Data Availability Statement

The original contributions presented in the study are included in the article/supplementary material, further inquiries can be directed to the corresponding author/s.

## Ethics Statement

Ethical review and approval was not required for the study on human participants in accordance with the local legislation and institutional requirements. Written informed consent for participation was not required for this study in accordance with the national legislation and the institutional requirements.

## Author Contributions

All authors listed have made a substantial, direct and intellectual contribution to the work, and approved it for publication.

## Funding

This study was supported by the Taichung Armed Forces General Hospital (Grant No.: TCAFGH-D-109011).

## Conflict of Interest

The authors declare that the research was conducted in the absence of any commercial or financial relationships that could be construed as a potential conflict of interest.

## Publisher's Note

All claims expressed in this article are solely those of the authors and do not necessarily represent those of their affiliated organizations, or those of the publisher, the editors and the reviewers. Any product that may be evaluated in this article, or claim that may be made by its manufacturer, is not guaranteed or endorsed by the publisher.
